# Multiscale micro-architecture of pore space in rocks: size, shape, deformation and accessibility determined by small-angle neutron scattering (SANS)

**DOI:** 10.1140/epje/s10189-023-00336-0

**Published:** 2023-09-08

**Authors:** Andrzej P. Radlinski, Tomasz Blach

**Affiliations:** 1 Faculty of Physics, University of Warsaw, Ludwika Pasteura 5, 02-093 Warsaw, Poland; 2 Queensland Micro Nanotechnology Centre, Griffith University, Nathan, Q4111 Australia

## Abstract

A brief summary of the evolving applications of small-angle neutron scattering (SANS) to the microstructural research on geological materials in the last few decades is provided, including new developments and possible future directions. This is an account of authors’ view of the interplay between the technical development of SANS instrumentation, methodology and sample environments and the progress of research on the evolution of organic matter, gas adsorption and desorption, fluid transport in the pore space and the microstructure of rocks, based mostly on their own research interests.

**Graphical abstract:**

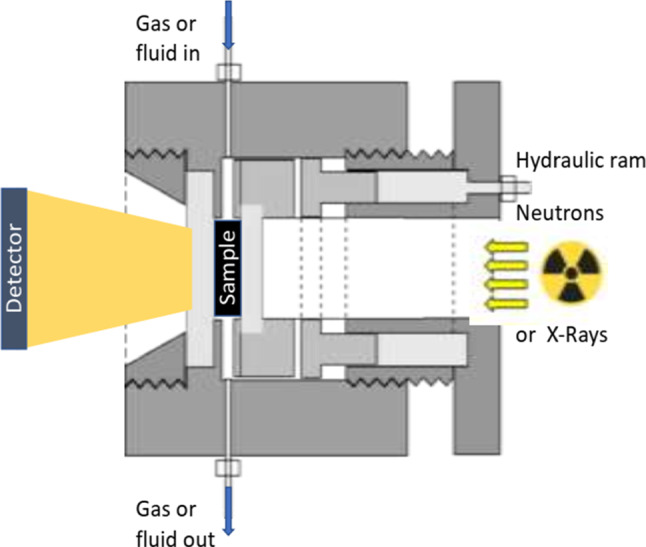

## Background

Porosity is the defining feature of Earth’s crust. Humanity’s economy and terrestrial life itself depends upon fluids hosted in the pores—gas, oil and, above all, the surface and subsurface water. Water present in the pore space originally served as a carrier (streams and rivers) and depositional environment (lakes and shallow seas) for the remnants of weathered rocks and living organisms (plants and bacteria). Energy-providing hydrocarbons are generated by molecular-scale chemical decomposition of compressed organic debris, which occurs at depth at elevated temperatures, and which migrate through the connected network of pores. The quantity and accessibility of those fluids is controlled by the geometry (total porosity) and topology (connectivity) of the pore space and the molecular-scale interactions between those fluids and the organic and inorganic components of the rock matrix (wettability, adsorption and desorption). Scales involved in various aspects of fluid retention and migration vary from molecular (sub-nanometres) to industrial (km) and planetary (1000 s of km).

The interface between the rock matrix and pore space in sedimentary rocks is rough over at least seven orders of magnitude of the linear scale, from sub-nanometres to centimetres; the roughness of landscape relief extends to the planetary scale. Since the early 1980’s, pore-matrix roughness has often been described using the framework of fractal geometry [1–9] and the mathematical formalism for two-phase systems using the correlation function [10]. Owing to wide applicability of the two-phase approximation for interpretation of SANS results acquired for geological materials, this formalism has been applied to successfully model the power-law SANS and SAXS results for many types of sedimentary rocks (pioneering work by Wong et al. [11] for shale, sandstones, limestone and dolomites, Bale and Schmidt [12] for coal and later work, e.g. [13–16]). P.W. Schmidt first established connection between the pore-size distribution (as an alternative expression of roughness) and the power-law dependence of the small-angle scattering intensity [17, 18]; within a decade the vast world of fractals (with non-universal fractal dimension) in natural porous media was revealed.

## SANS as a tool for microstructural research in geo-materials

SANS is uniquely suited to microstructural geological applications due to its capacity to provide volume-average pore-size-specific nano- and microstructural information, high penetration power of neutrons and sensitivity to the isotopic composition of the pore content (e.g., [13, 14, 19–23] and references therein). The sister SAXS technique has been used less extensively (e.g., [15, 24–26]). In the early days, the crucial issue was the extent of the experimentally accessible Q-range (hence the range of corresponding pore sizes); the capacity to study pore sizes larger than several hundred nanometres was determined by the small-Q limit (of about 1 × 10^−3^ Å^−1^) of the longest-base pinhole SANS instrument (D11 at ILL for the last 50 years [27]). The experimentally accessible range was then greatly improved in 1997, following the construction of a Bonse-Hart type USANS instrument by M. Agamalian [28]. Since that time, pore sizes ranging from sub-nanometres to approximately 20 µm can in principle be investigated using a combination of absolutely calibrated SANS and USANS results [29].

SANS and USANS results from strongly scattering rocks are easily contaminated by multiple scattering (MS). The capability of long base SANS instruments (using wavelengths of ca. 4–5 Å) to provide overlap with USANS data (using 1.5 Å < λ < 3 Å; e.g., [30, 31]) in the region around Q≈10^–3^ Å^−1^ has been crucial to the elimination of MS from free-standing oriented sub-millimetre rock samples with thickness larger than ca 0.1 mm. MS is specific to strong scatterers (like most rocks) and affects various extended Q-range SANS instruments (e.g., VSANS, MSANS and SAMBA) as well as the time-of-flight (TOF) SANS instruments, which all rely on data collected at longer wavelengths, up to 20 Å [29, 32–35]. Representativity of the size and form of samples is a contentious issue due to the pervasive multi-scale heterogeneity of rocks. Various approaches to sample preparation for SANS and USANS measurements include gently crushed coarse powders (sieved to a required grain size to represent an average of a larger rock volume) or solid samples cut in different orientations to bedding, e.g. [36]; chosen thicknesses of samples vary from 0.2 to 1 mm [37] to about 50 μm [21], depending on the microstructural aspect studied. The precedent (and not well examined) issue is spatial uniformity of rocks formed in similar depositional environments on the centimetre linear scale. It has been recently studied for shale cores [37] as a fundamental question where several adjacent samples are used and the results are interpreted jointly (e.g., for destructive microstructural measurements using high uniaxial pressure [38] or a chemically reactive environment [39]).

## Microstructural models

The great majority of sedimentary rocks are two-phase (pore-matrix) systems owing to the isotopic composition of most abundant minerals [14, 22]. This facilitates use of microstructural models based on a polydisperse distribution of spheres [19, 40] or other methods, including maximum entropy [41]. These approaches have been widely used, especially for analysis of azimuthally isotropic SANS results obtained from coarse powders or solid samples orientated in-bedding. The major quantitative characteristics computed using these models include the total porosity, specific surface area and pore size distribution.

The vertical—horizontal anisotropy, however, is an inherent feature of sedimentary rocks. Moreover, the pore shape anisotropy may be size specific [36]. SANS can only provide volume-averaged general information on pore anisotropy [42–45]. Quantitative analysis of anisotropy has been rarely performed, with exception of recent work on tight rocks: Gu et al. [46] demonstrated different anisotropy for pores hosted in the inorganic and organic component of the rock matrix and Blach et al. [47] have shown that there is a correlation between the regions of hydrocarbon generation and pore anisotropy.

## Contrast matching and measurements of pore accessibility

The reorientation towards the “green economy” posed new challenges for microstructural research on sedimentary rocks. Growing interest in the subsurface storage of greenhouse CO_2_ in rock formations, industrial production of the coal-bed methane and the extraction of natural gas from tight (unconventional) shales as an interim energy source has led to the gradual development of various SANS environmental gas cells capable of simulating the subsurface pressure and temperature conditions using methane (and d-methane), CO_2_ (gas and liquid), helium and various aqueous solutions (references [22] (chapter 4.2) and [25, 48–51]). The option of using gases pressurised to more than 100 MPa (1 kbar), especially greenhouse gases, offers significant improvements to geoscientific applications of contrast matching (CM) compared to the “classical” use of less-penetrating deuterated liquids (e.g., heavy water and deuterated heavy hydrocarbons [51–53]). This development made it possible to separately characterise the total porosity, specific surface area and pore size distribution for porous space that is accessible or inaccessible to penetrating fluids. Such results are of particular interest for tight unconventional gas reservoirs, where the accessible porosity is concentrated mainly in the nanopores and often constitutes a small fraction of total porosity [23, 54, 55].

Significantly, for a great majority of analysed rocks the roughness of the pore—matrix interface for the accessible pores turned out to be less accentuated than that for the inaccessible pores. This provides an interesting insight into the long-standing fundamental question of the origin and temporal evolution of the local morphology in rocks; it appears that in addition to the anti-sintering mechanism of pore generation, which is driven by the interplay between the grain—grain and grain—pore content free energy (proposed by Cohen [56]), a significant role may be played by reactive transport of acidic formation fluids through the pore space [57–60]. Consequently, the fractal geometry of rocks is characterised not by one (e.g., [12, 12, 29]) but two different non-universal surface fractal dimensions, D_s_: one for the accessible pore space and the other for the inaccessible pore space, where 2 ≤ D_s_(accessible) < D_s_(inaccessible) ≤ 3.

## Condensation of greenhouse gases in nanopores and sub-nanopores

Technical developments resulting in low background noise of modern SANS detectors have enabled precise measurements of neutron scattering in the large-Q region (Q > 0.1 Å^−1^), which corresponds to the nano- and sub-nanometre size pores (e.g., [61]). These results have helped to gain insight into the ubiquitous phenomenon of capillary condensation in the nano-pores of shales and carbonates (e.g., [23, 51, 54]. Similar observations have been made for coal (e.g., [25, 48, 61]) and aerogels [62] (considered as simple man-made analogues of natural rocks: e.g., hydrogen in C-aerogel [63], CO_2_ in silica aerogels [64] and methane in Si-aerogel [65]). It turned out that the rock-specific density of greenhouse gases in nano-confinement often exceeds the bulk density of the pressurised gas by a factor of two to three and much more for aerogels. The large number density of nanopores in shales makes nanopores a major reservoir of absorbed methane and/or carbon dioxide in geological formations.

## Deformation of the pore space under uniaxial stress

The recently added uniaxial stress capability of high pressure environmental cells enabled SANS and USANS measurements under simulated pressure conditions similar to those encountered in unconventional shales subjected to hydraulic fracturing. Porosity response to the in-situ-like hydrostatic stress cycling [51] and a combination of hydrostatic and uniaxial stress cycling [38] is complex: it depends on the pore size and differs for rocks with different thermal maturity of organic matter. The stress-induced structural modification of the accessible (and inaccessible) fraction of the pore space is significant, irreversible and its extent strongly depends on the details of pressure cycling. These results demonstrate that an industrial well stimulation procedure is a fine-tuned irreversible process, in particular on the scales from sub-nanometres to tens of micrometres which control the adsorption, desorption and primary migration of methane in the subsurface.

## Conclusion and future developments

In recent years the small-angle neutron scattering techniques (SANS and USANS) have become a mainstream research tool in petrology and geology on the nano- and microscale. In the last decade it has been used as the preferred non-invasive analytical method for research on the pore-size-specific micro- and nanoporosity (accessible and inaccessible to penetrating fluids), complementary to the established methods of microscopy (optical, SEM and TEM), gas adsorption measurements and mercury intrusion porosimetry. Further development of the high-pressure (hydrostatic, uniaxial and tri-axial) and high-temperature environmental capabilities (to the pressures in excess of 400 MPa and temperatures of the 500 °C range, compared to the currently certified *p*_max_ = 120 MPa and *T*_max_ = 100 °C) will enable SANS measurements in simulated geological conditions corresponding to much greater depths. A relatively new capacity is the option of in-situ measurement of interactions with various fluids, including time-resolved observation of the evolution of the pore space during reactive flow.

Extension of the non-invasive structural observations from ca. 10 µm (the upper limit of the USANS technique) to the centimetre scale has been recently enabled by development of the neutron [66] and combined neutron/X-ray tomographic equipment [67] and the corresponding instrument compatibility modifications made to the SANS environmental cell. Combined SANS/USANS/tomography measurements provide structural data extending over 10 orders of magnitude (from sub-nanometres to centimetres) with an option of contrast adjustment and kinetic studies in controlled pressure and temperature conditions (including cryogenics). Potential applications of such research tools extend well beyond Earth Sciences, to the fields of materials engineering, hydrogen storage, chemistry, biology, medical research and industrial applications, to name a few.

## Data Availability

Data sharing is not applicable to this article as no datasets were generated or analysed during the current study.
